# Supplementary material for: Comparative proteomic profiling of receptor kinase signaling reveals key trafficking components enforcing plant stomatal development
Source: Sci Adv. 2026 Mar 25;12(13):eaeb6464. doi: 10.1126/sciadv.aeb6464 (PMC13015889; doi:10.1126/sciadv.aeb6464)
Supplement: Supplementary file 1 — Figs. S1 to S13 Tables S1 and S2 Legend for movie S1 Legends for data S1 to S5 [file sciadv.aeb6464_sm.pdf]

Science Advances
AAAS

Supplementary Materials for

Comparative proteomic profiling of receptor kinase signaling reveals key trafficking components enforcing plant stomatal development

Pengfei Bai *et al.*

Corresponding author: Keiko U. Torii, ktorii@utexas.edu

Sci. Adv. **12**, eaeb6464 (2026)
DOI: 10.1126/sciadv.aeb6464

The PDF file includes:

Figs. S1 to S13
Tables S1 and S2
Legend for movie S1
Legends for data S1 to S5

Other Supplementary Material for this manuscript includes the following:

Movie S1
Data S1 to S5

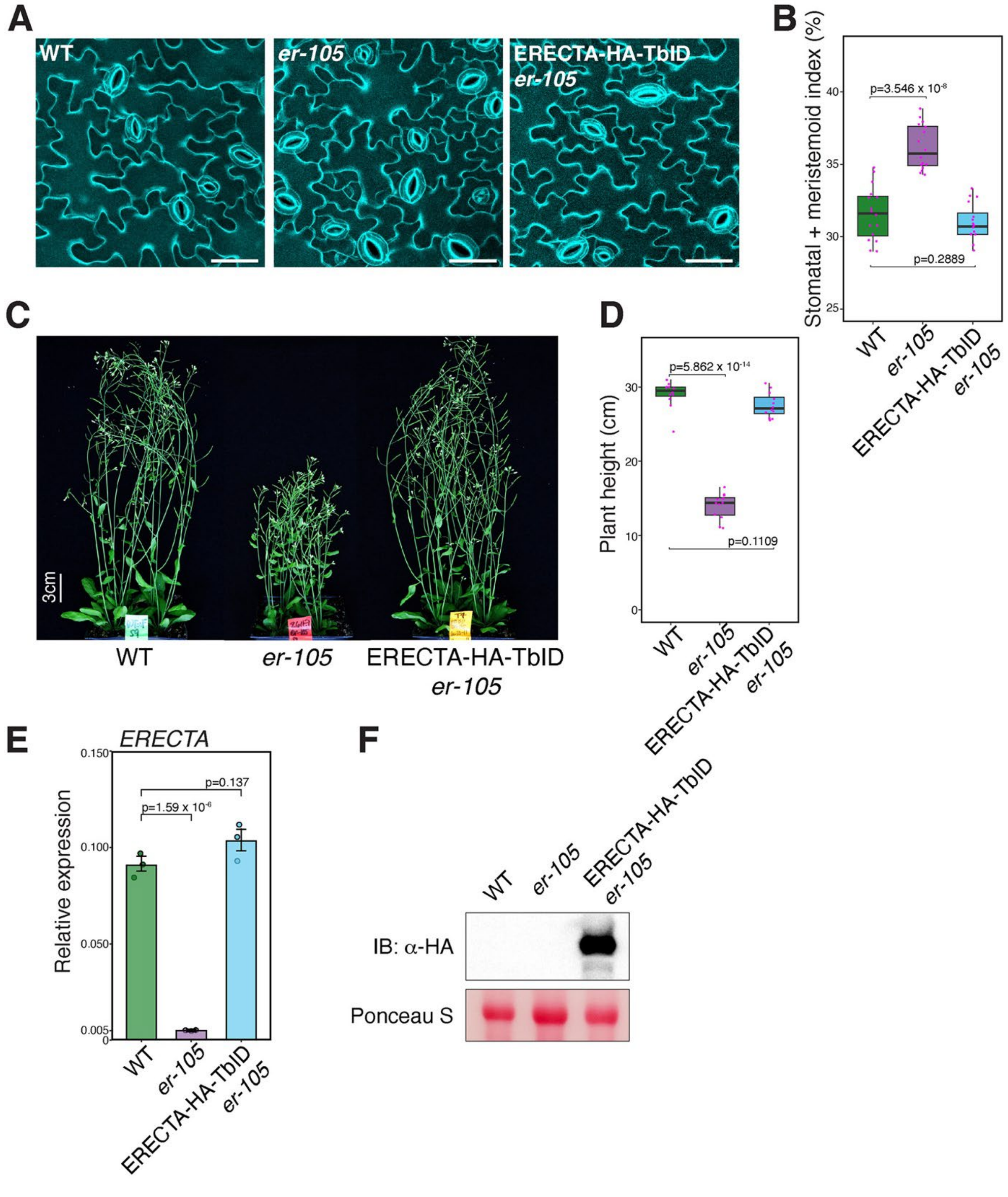

Fig. S1. ERECTA-HA-TbID transgene rescues the stomatal and morphological defects of the *er-105* mutant.

(**A**) Confocal images of abaxial epidermis from WT, *er-105*, and ERECTA-HA-TbID complementation lines in the *er-105* background. Expression of ERECTA-HA-TbID restores defects of stomatal number and patterning observed in *er-105* mutants. Scale bars: 40 µm.

(**B**) Quantification of stomatal and meristemoid index. ERECTA-HA-TbID significantly rescues the elevated stomatal and meristemoid index phenotype of *er-105*. Statistical analysis was performed using one-way ANOVA followed by Tukey's HSD test. n = 12 independent cotyledons from distinct seedlings.
(**C**) Representative images of plant height at 5 weeks old. The dwarf phenotype of *er-105* is fully rescued in ERECTA-HA-TbID transgenic plants, resembling WT morphology. Scale bar: 3 cm.
(**D**) Quantification of plant height shows significant recovery in complementation lines compared to *er-105* mutants. Statistical analysis was performed using one-way ANOVA followed by Tukey's HSD test, with significant differences (p-values) noted for specific genotype comparisons (n = 12 independent plants).
(**E**) Relative expression of *ERECTA* measured by RT-qPCR in WT, *er-105*, and *ERECTA-HA-TbID* complementation lines. *ERECTA* transcript levels are restored in the complementation lines to near WT levels. Expression levels are normalized to ACT2 and shown relative to WT. Error bars indicate standard errors (n = 3 replicates).
(**F**) Immunoblot showing expression of ERECTA-HA-TbID fusion protein. α-HA detects HA-tagged ERECTA; Ponceau S staining indicates equal protein loading.

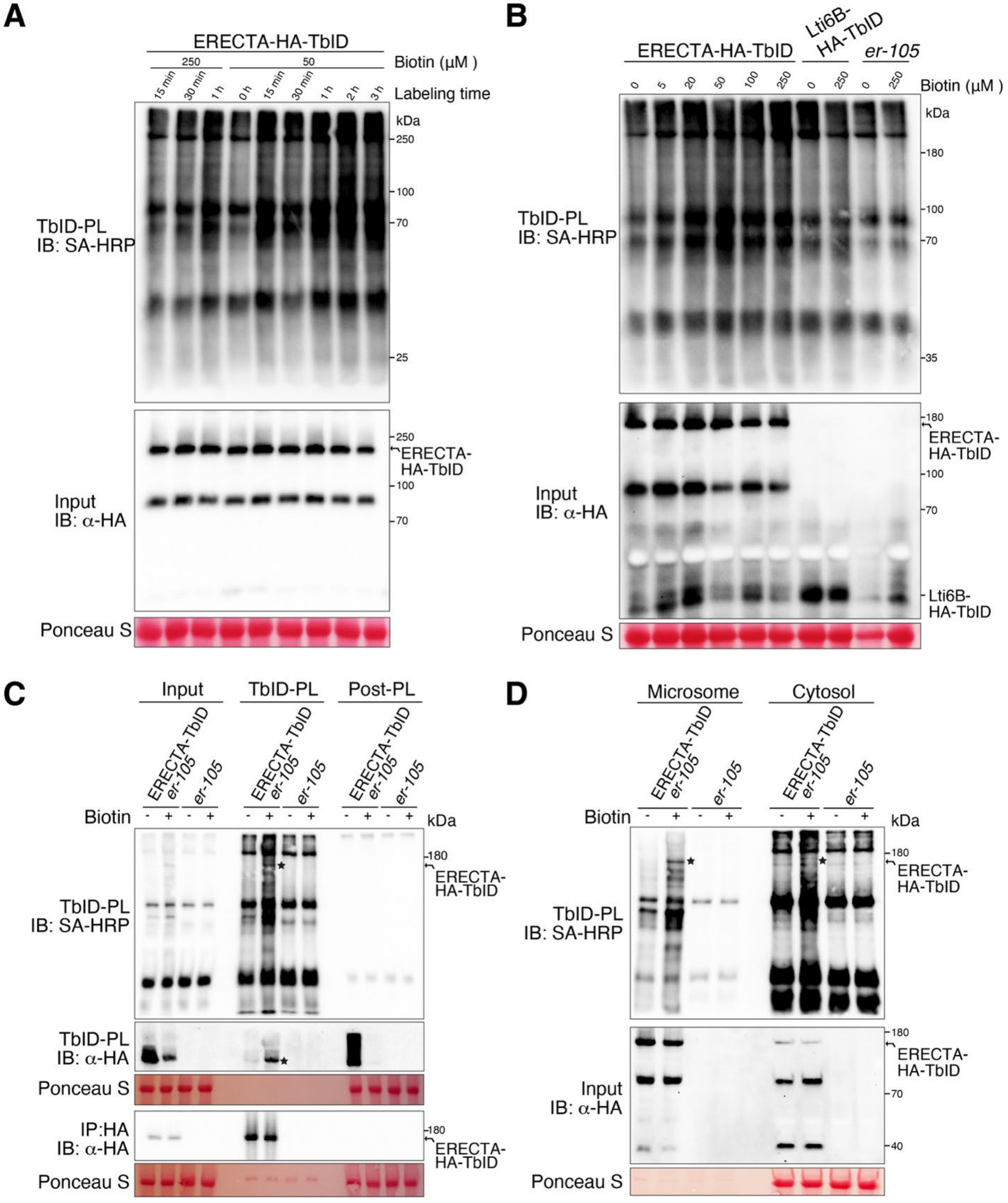

Fig. S2. Optimization of ERECTA-TbID proximity labeling conditions.
(**A**) Time-course and dose-response analysis of biotin treatment (50 μM and 250 μM, 15 min to 3 h) in ERECTA-HA-TbID seedlings. SA-HRP blot (top) shows increased biotinylation over time.

α-HA blot (middle) confirms comparable levels of ERECTA-HA-TbID. Ponceau S (bottom) shows loading control.

(**B**) Biotin titration from 0 to 250 μM in ERECTA-HA-TbID and controls (Lti6B-HA-TbID and *er-105*). SA-HRP blot (top) shows dose-dependent labeling efficiency. α-HA blot (middle) confirms the levels of ERECTA-HA-TbID. Ponceau S staining (bottom) serves as loading control.

(**C**) Immunoblot validation of optimized TbID-PL. SA-HRP blot (top) and α-HA blot (middle) show robust biotinylation of proximal proteins and enrichment of ERECTA-HA-TbID, respectively. Post-PL fractions confirm depletion of biotinylated proteins. Ponceau S staining indicates loading controls.

(**D**) Subcellular fractionation of ERECTA-TbID proteomes reveals compartment-specific enrichment. Microsomal and cytosolic fractions were analyzed by SA-HRP blot (top) and α-HA blot (middle), showing enriched biotinylation in microsomal fractions with reduced background.

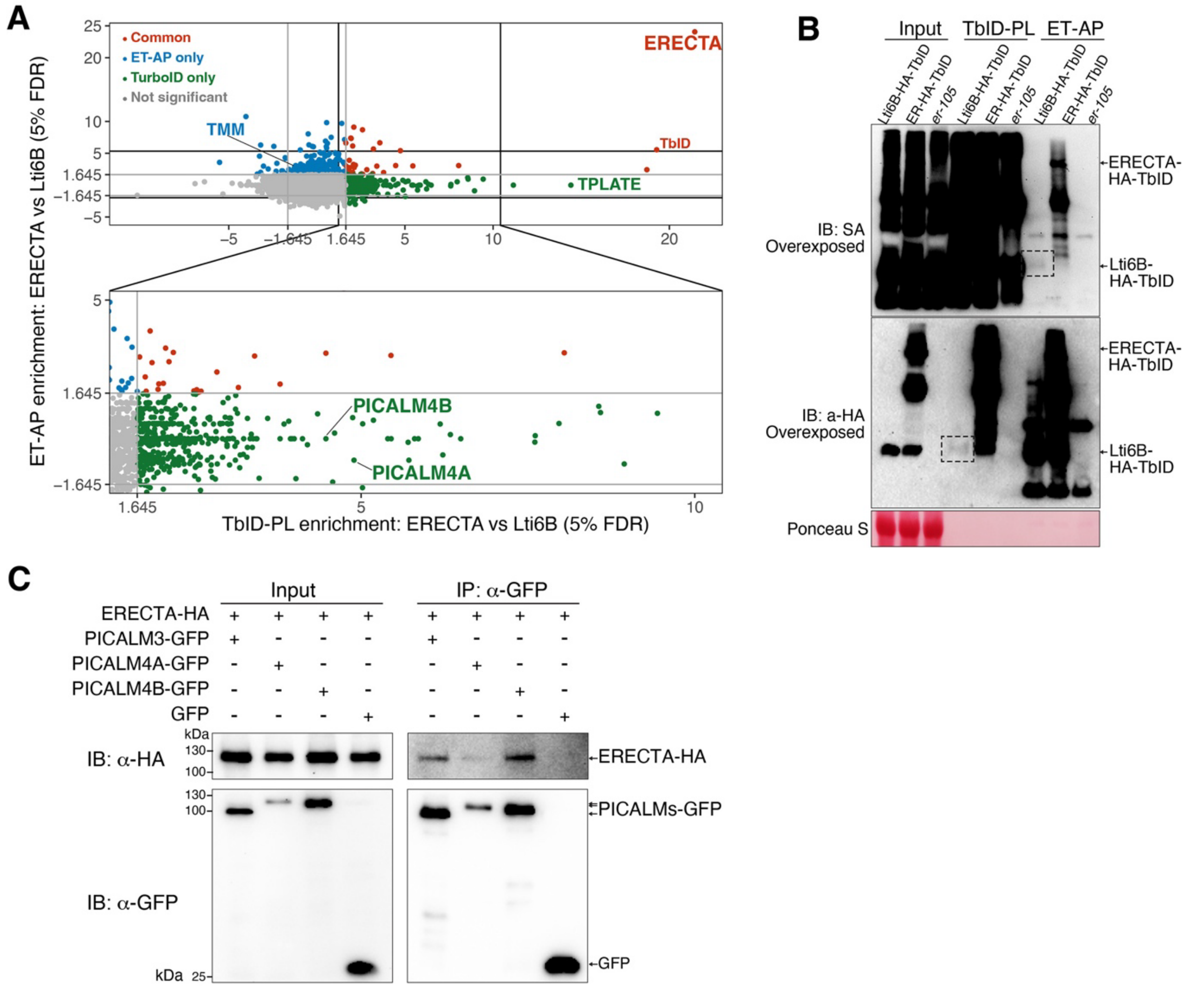

Fig. S3. Proteomic and biochemical validation of ERECTA-PICALM associations.

(**A**) Comparative enrichment analysis of ERECTA-associated proteins by TbID-PL and ET-AP, using ERECTApro::Lti6B-HA-TbID as the negative control. A total of 601 proteins were detected by TbID-PL and 276 proteins by ET-AP, with 35 proteins shared between the two approaches. PICALM4A and 4B are preferentially enriched by TbID-PL, whereas TMM is detected exclusively by ET-AP, consistent with the findings in main Fig. 1E. All TbID-PL and ET-AP experiments were performed using three biological replicates. Protein enrichment was quantified as log2-transformed ratios of normalized PSMs between experimental and control conditions and significance was assessed using a one-sided Z-test ($z \geqslant 1.645$, BH-adjusted FDR). Proteins enriched in both approaches are shown in red, TbID-PL specific enrichments in green, and ET-AP specific in blue.

(**B**) Overexposed immunoblot highlighting weak accumulation of the ERECTApro::Lti6B-HA-TbID negative control. Both streptavidin-HRP (SA-HRP) blot (top) and α-HA immunoblot (middle) confirm the presence of positive biotinylated, but extremely low-abundance Lti6B-HA-TbID proteins.

(**C**) Transient protoplast co-immunoprecipitation assays validating ERECTA interactions with multiple PICALM proteins. Arabidopsis protoplasts were co-transfected with *ERECTA-HA* and

GFP tagged *PICALM3, 4A, 4B*. Immunoblot analysis confirmed *in vivo* binding of ERECTA-HA to PICALM3-GFP, PICALM4A-GFP, and PICALM4B-GFP, but not with GFP alone.

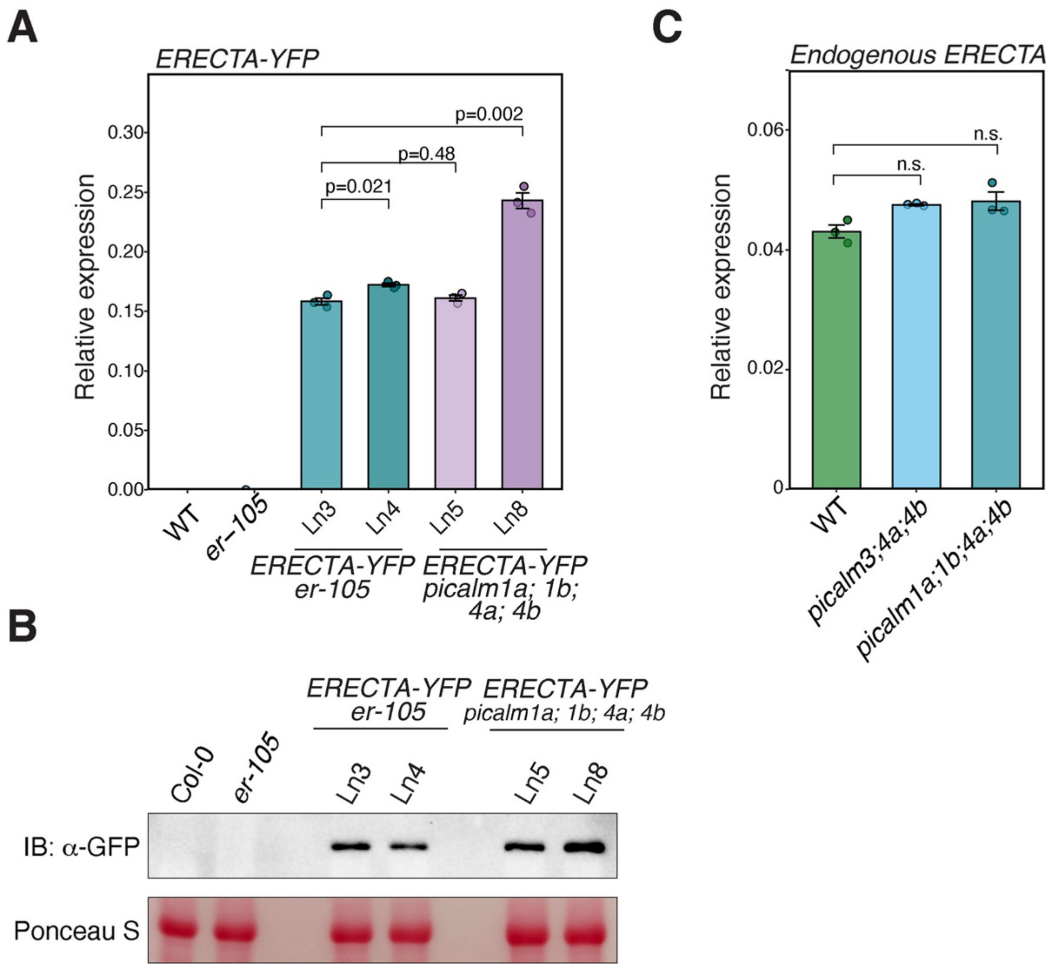

Fig. S4. Expression analysis of *ERECTA-YFP* transgenes and endogenous *ERECTA* in *picalm* mutant backgrounds.

(**A**) Relative expression of the *ERECTA-YFP* transgene measured by RT-qPCR. Independent transgenic lines expressing *ERECTA-YFP* in the *er-105* background (Ln3, Ln4) and in the *picalm1a;1b;4a;4b* background (Ln5, Ln8) were analyzed. Ln3 in the *er-105* background and Ln5 in *picalm1a;1b;4a;4b* background exhibit comparable *ERECTA-YFP* transcripts and were used in subsequent experiments. Expression levels are normalized to *ACT2* and error bars represent standard errors (n = 3 replicates).

(**B**) Immunoblot analysis of ERECTA-YFP protein accumulation in the same transgenic lines. α-GFP detects ERECTA-YFP; Ponceau S staining represents total protein loading amount.

(**C**) Relative expression of endogenous *ERECTA* in WT, *picalm3;4a;4b* and *picalm1a;1b;4a;4b*. RT-qPCR measurement demonstrates no significant changes (n.s.) in endogenous *ERECTA* transcript levels across these genotypes. Expression levels are normalized to *ACT2* and error bars represent standard errors (n = 3 replicates).

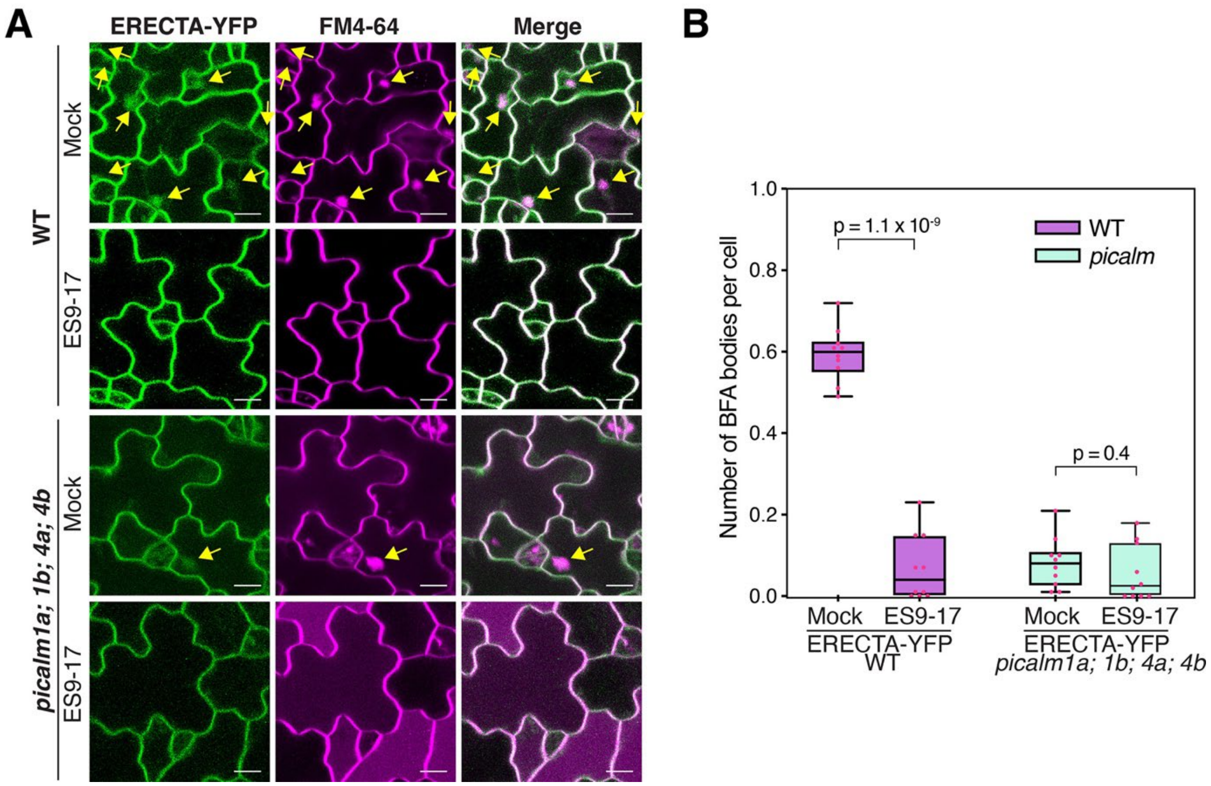

Fig. S5. Loss of PICALMs renders ERECTA endocytosis insensitive to the CME inhibitor ES9-17.

(**A**) Confocal images showing ERECTA-YFP internalization in WT and *picalm1a;1b;4a;4b* mutant seedlings (4-day-old) treated with mock (DMSO) or 100 µM ES9-17 for 1 h. In WT, Brefeldin A (BFA) induces the formation of ERECTA-YFP-positive endosomal compartments (BFA bodies, arrowheads) under mock conditions, whereas ES9-17 markedly reduces the number of BFA bodies, indicating inhibition of clathrin-mediated endocytosis (CME) of ERECTA. In contrast, the *picalm1a;1b;4a;4b* mutant shows no significant difference in BFA body formation between mock and ES9-17 treatments, indicating that CME activity is disrupted in the mutant background. Scale bar: 10 µm

(**B**) Box plot quantification of ERECTA-YFP positive BFA bodies per cell in WT and *picalm1a;1b;4a;4b* mutant under mock and ES9-17 treatments. Statistical significance was determined using Welch's two-sample t-test (n = 10 independent seedlings).

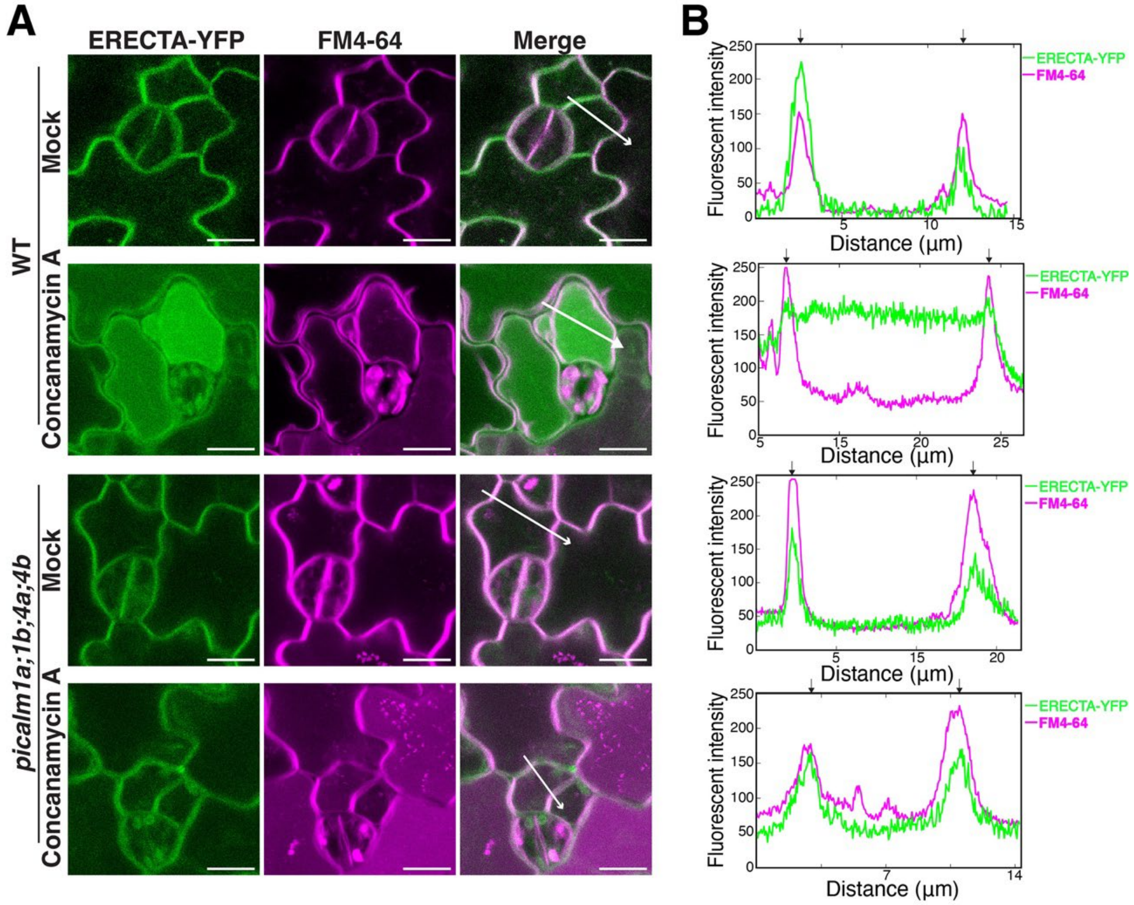

Fig. S6. Inhibition of vacuolar acidification by Concanamycin A reveals that endocytosed ERECTA-YFP by PICALMs is eventually trafficked to the lytic vacuole.

(**A**) Representative confocal images showing ERECTA-YFP (green) and FM4-64 (magenta) in the abaxial epidermis of cotyledons from WT and *picalm1a;1b;4a;4b* mutants after 1 µM Concanamycin A treatment. Seedlings were pre-stained with 5 µM FM4-64 for 30 min, rinsed and incubated in water for 6 h to allow FM4-64 to traffic to endosomes and the tonoplast. Seedlings were subsequently vacuum infiltrated with 1 µM Concanamycin A and incubated for an additional 6 h. In WT, Concanamycin A induces strong accumulation of ERECTA-YFP signals within the lytic vacuole, consistent with trafficking patterns previously reported for ERL1. In contrast, Concanamycin A treatment does not enrich vacuolar ERECTA-YFP signals in *picalm1a;1b;4a;4b* mutant. Scale bars: 10 µm.

(**B**) Line-scan fluorescence intensity profiles of ERECTA-YFP (green) and FM4-64 (magenta) along the white arrows in (A). Black arrows mark FM4-64 staining position at the tonoplast, the distance along each plot corresponds to the length of the white arrows in (A).

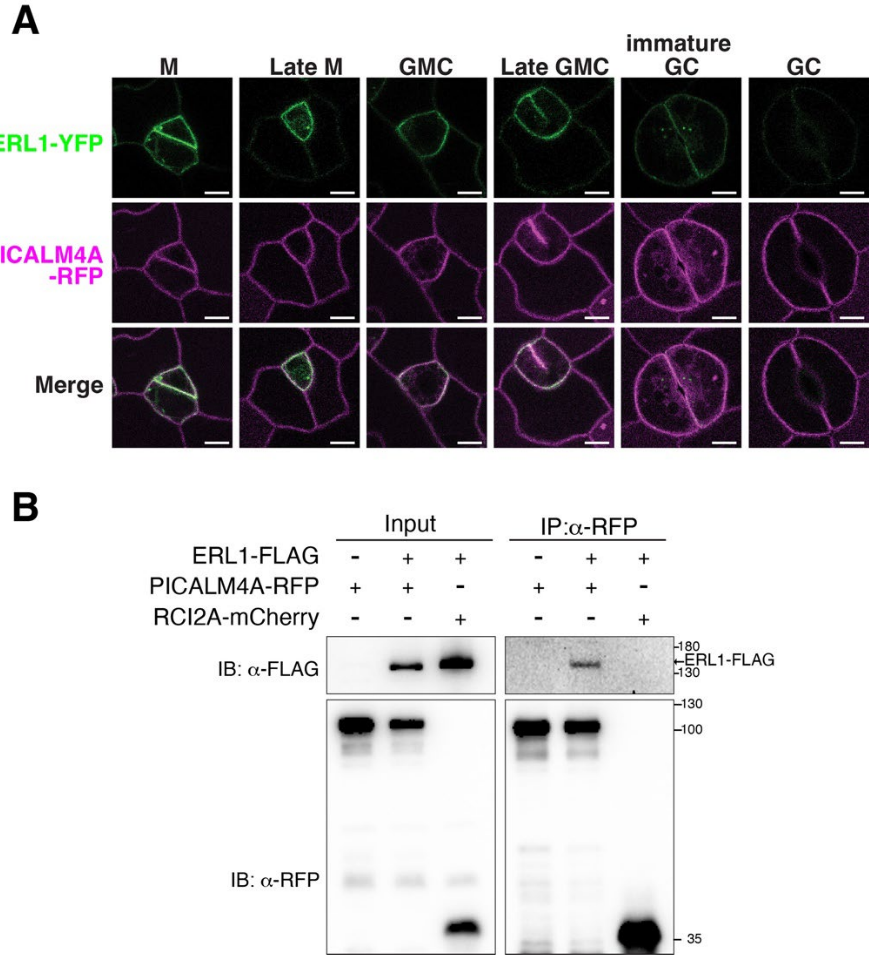

Fig. S7. ERL1-YFP colocalizes and interacts with PICALM4A-RFP *in vivo*.
(**A**) Confocal images of Arabidopsis epidermal cells expressing ERL1-YFP (green) and PICALM4A-RFP (magenta) across defined stages of stomatal development, including meristemoid (M), late meristemoid (Late M), guard mother cell (GMC), late GMC, immature guard cell (immature GC), and mature guard cell (GC). ERL1-YFP colocalizes with PICALM4A-RFP at the plasma membrane during early stages and at the cell plate in dividing GMCs (e.g., Late GMC), suggesting stage-specific colocalization of PICALM adaptors with ERL1. Scale bars: 10 µm.
(**B**) Co-immunoprecipitation (Co-IP) assay showing that ERL1 interacts with PICALM4A *in vivo*. Transgenic Arabidopsis lines expressing both *ERL1-FLAG* and *PICALM4A-RFP* under their native promoters were subjected to Co-IP experiments by α-RFP immunoprecipitation. ERL1-FLAG was detected in the PICALM4A-RFP IP fraction, demonstrating the positive association between ERL1 and PICALM4A. For the original, uncropped gel blot images, see fig. S13.

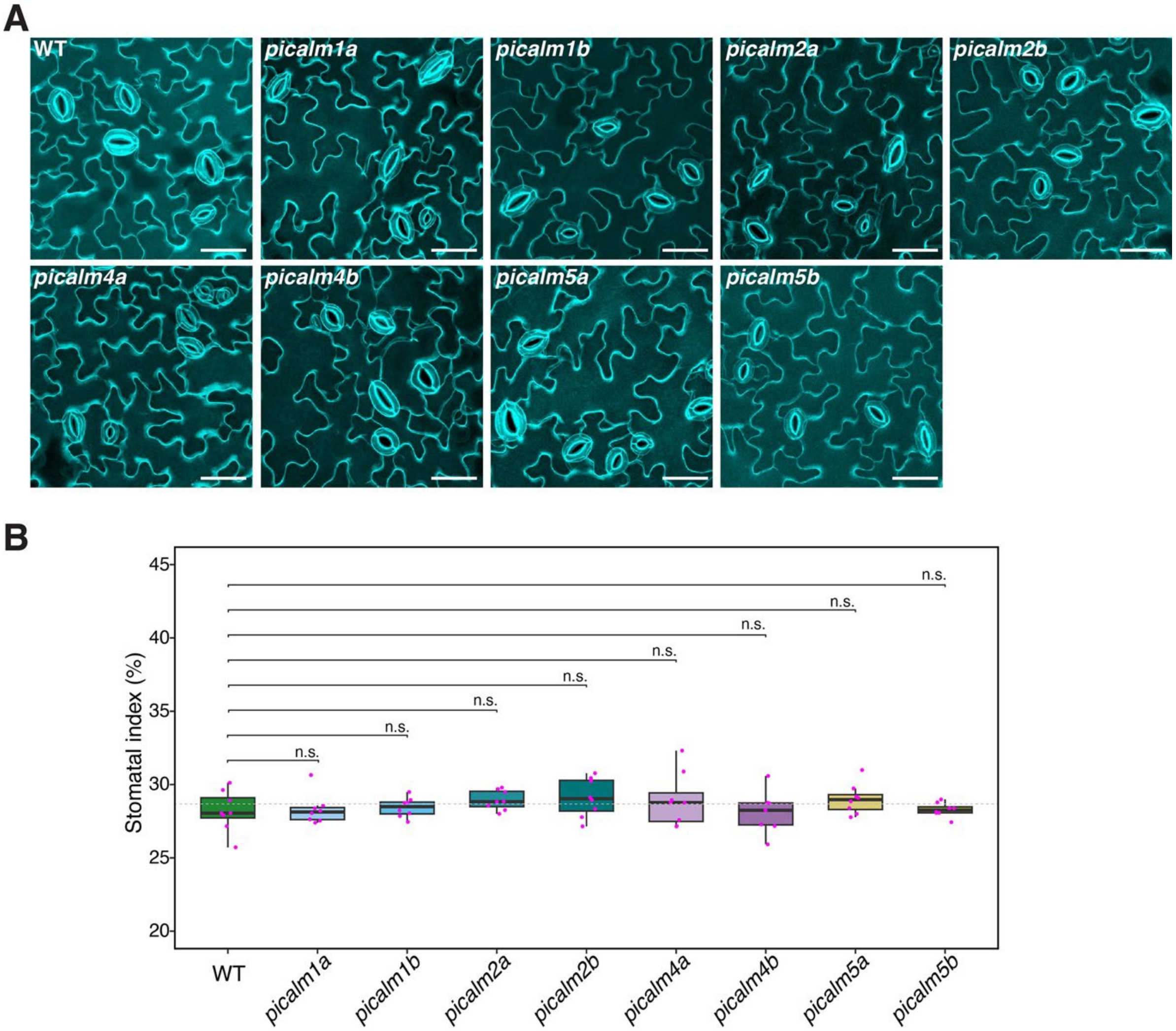

Fig. S8. Single mutants of *PICALM* genes confer no discernible phenotype on stomatal development.

(**A**) Confocal images of abaxial leaf epidermis from WT and single *picalm* mutants (*picalm1a*; *picalm1b*, *picalm2a*, *picalm2b*, *picalm4a*, *picalm4b*, *picalm5a*, and *picalm5b*). Loss of individual *PICALM* genes does not affect stomatal patterning. Scale bars: 40 μm.

(**B**) Quantification of stomatal index in WT and single *picalm* mutants; no significant changes (n.s.) were detected between WT and these tested single *picalm* mutants, as determined by one-way ANOVA followed by Tukey's HSD test. n = 8 independent cotyledons from distinct seedlings.

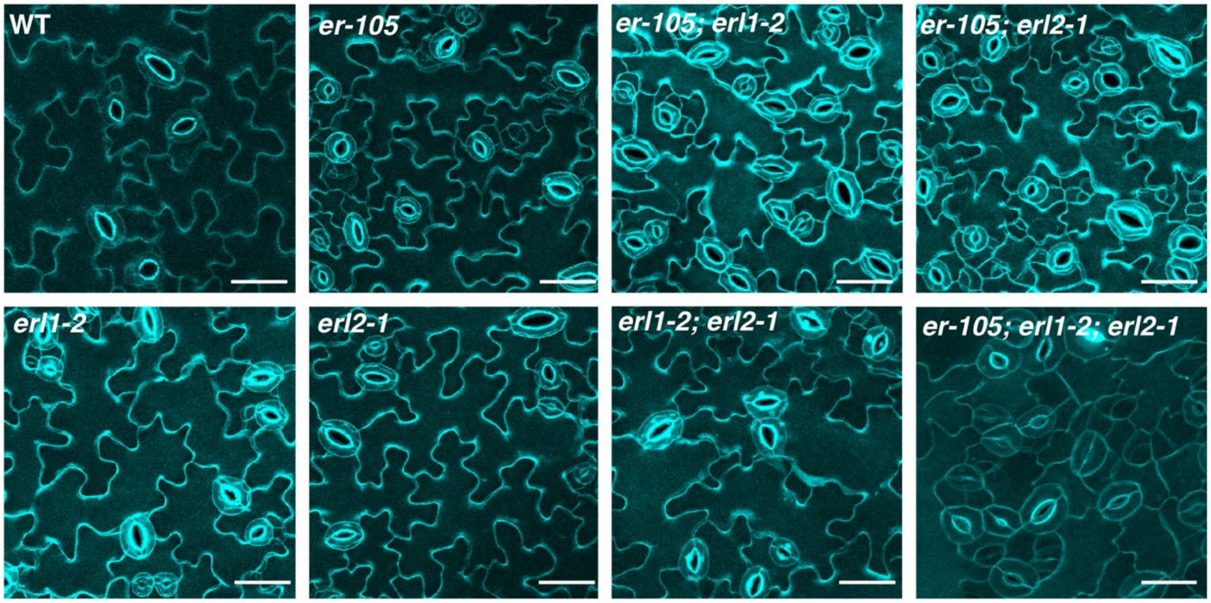

Fig. S9. Stomatal patterning defects in *ERECTA*-family mutants.

Confocal images of abaxial leaf epidermis from WT and various combinations of *ERECTA* family single, double, and triple mutants. While single mutants (*er-105*, *erl1-2*, *erl2-1*) show mild to moderate defects in stomatal patterning, severe stomatal clustering and overproliferation are observed in double and triple mutants, especially in the *er-105; erl1-2; erl2-1* mutant. These results confirm the synergistic role of ERECTA-family receptors in maintaining stomatal patterning. Scale bars: 40 µm.

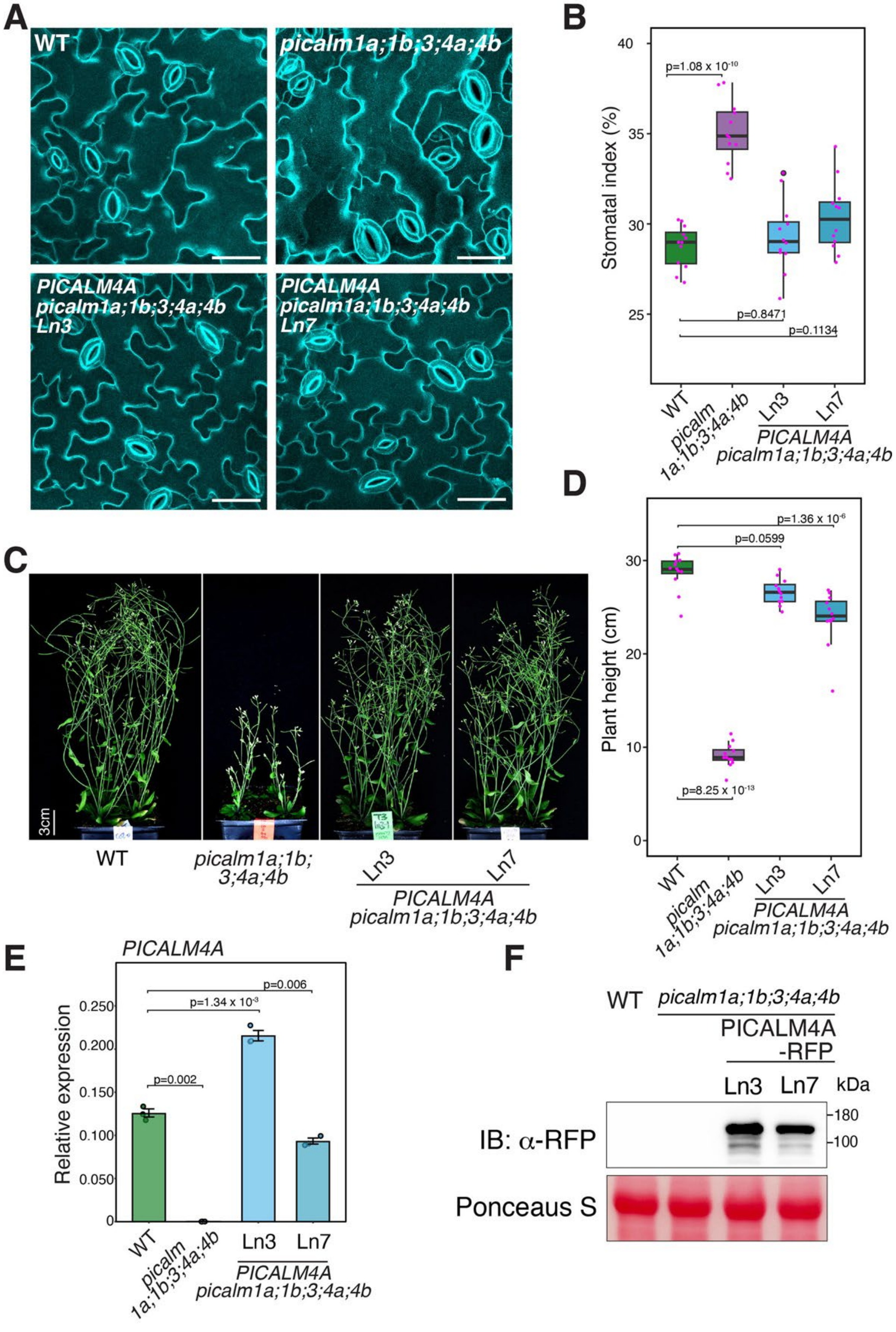

Fig. S10. PICALM4A is sufficient to rescue stomatal and growth defects in *picalm1a;1b;3;4a;4b* quintuple mutant.

(**A**) Confocal images of abaxial epidermis from WT, *picalm1a;1b;3;4a;4b* mutants, and two independent PICALM4A-RFP complementation lines (Ln3, Ln7). *PICALM4A* expression restores normal stomatal phenotype. Scale bars: 40 µm.
(**B**) Quantification of stomatal index. Complementation with PICALM4A-RFP significantly rescues the elevated stomatal index observed in the *picalm* mutant. Statistical analysis was performed using one-way ANOVA followed by Tukey's HSD test, with significant differences (p-values) noted for specific genotype comparisons. n = 12 independent cotyledons from distinct seedlings.
(**C**) Representative images of whole plants at 5 weeks old. Growth defects seen in *picalm1a;1b;3;4a;4b* are suppressed in PICALM4A-RFP complementation lines. Scale bar: 3 cm.
(**D**) Quantification of plant height. PICALM4A-RFP expression restores plant height to near wild-type levels. Statistical analysis was performed using one-way ANOVA followed by Tukey's HSD test, with significant differences (p-values) noted for specific genotype comparisons (n = 15 independent plants).
(**E**) Relative PICALM4A-RFP transcript levels measured by RT-qPCR in complementation lines Ln3 and Ln7. Expression levels are normalized to ACT2 and shown relative to WT. Error bars represent standard errors (n = 3 replicates).
(**F**) Immunoblot analysis of PICALM4A-RFP protein accumulation in Ln3 and Ln7 using α-RFP antibody. Ponceau S staining indicates equal loading.

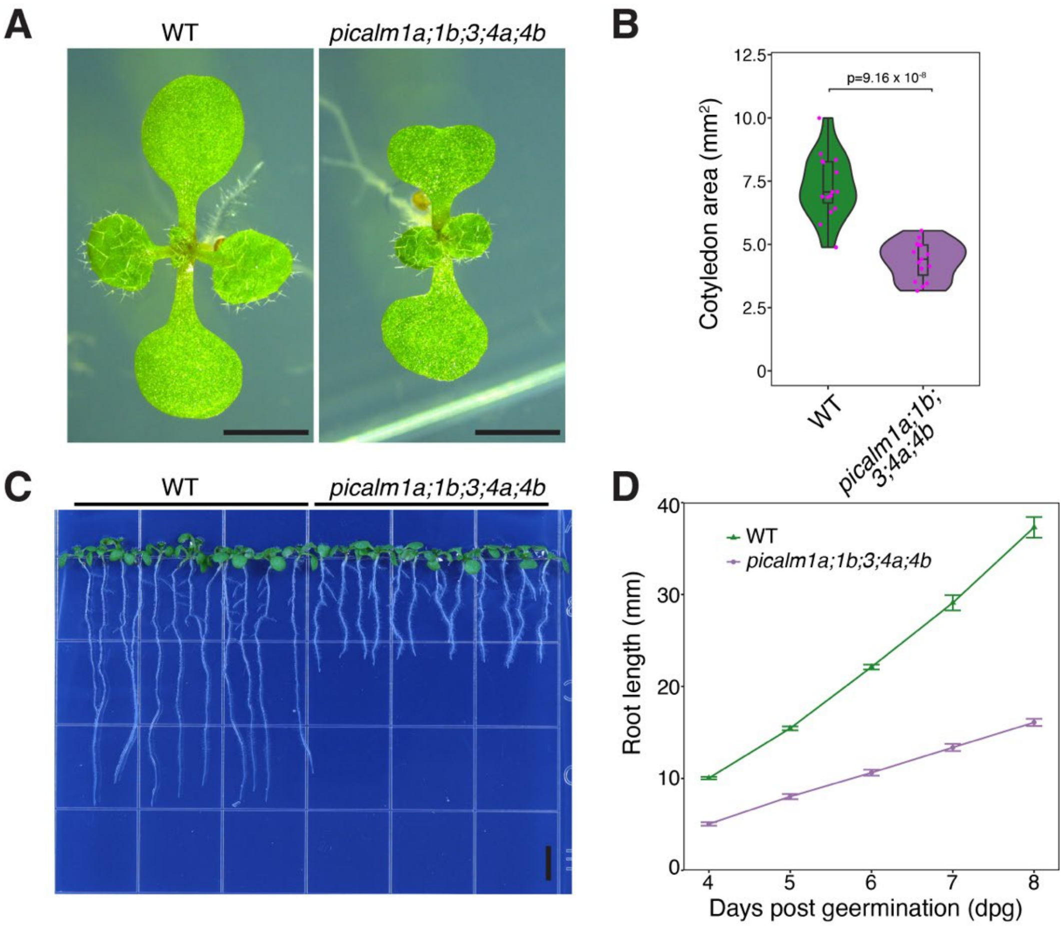

Fig. S11. The *picalm1a;1b;3;4a;4b* quintuple mutant exhibits early developmental defects.

(**A**) Representative images of WT and *picalm1a;1b;3;4a;4b* seedlings at 8 days post-germination (dpg). The *picalm1a;1b;3;4a;4b* mutant seedlings display smaller cotyledons. Scale bars: 2 mm.

(**B**) Quantification of cotyledon area in WT and *picalm1a;1b;3;4a;4b* seedlings at 8 dpg. The mutant shows significantly reduced cotyledon size. Violin and box plots display the distribution and interquartile range of cotyledon area measurements (n = 15 independent seedlings). Statistical significance was determined by Student's t-test.

(**C**) Root growth phenotype of WT and *picalm1a;1b;3;4a;4b* seedlings on vertical plates at 8 dpg. Mutants show reduced primary root elongation. Scale bars: 5 mm.

(**D**) Quantification of root length over time from 4 to 8 dpg. *picalm1a;1b;3;4a;4b* mutants show significantly shorter roots than WT. Data represent mean ± SE (n = 12 independent seedlings).

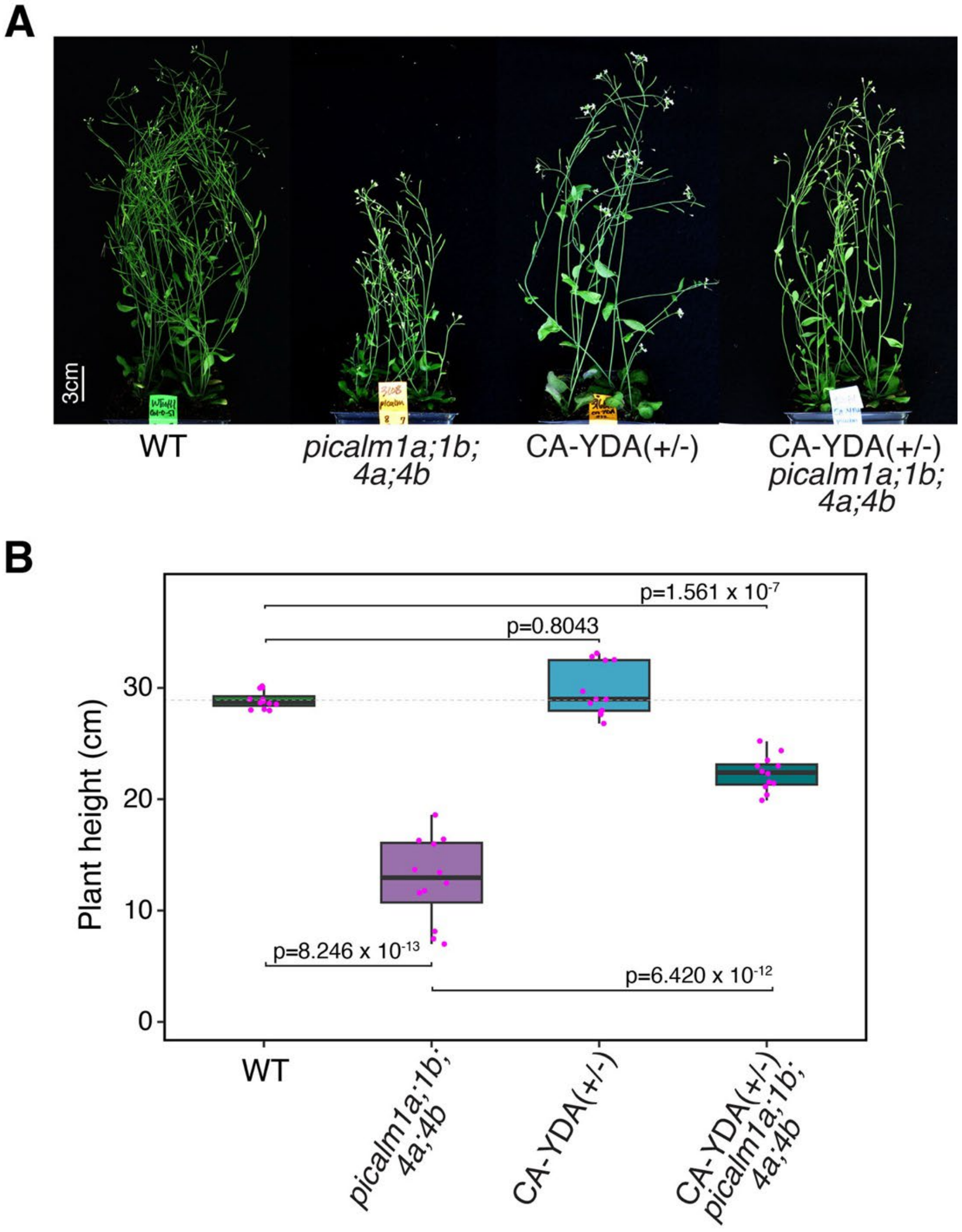

Fig. S12. Constitutively active YDA partially rescues the dwarf phenotype of higher-order *picalm* mutants.

(**A**) Representative images of plant height at 5 weeks old. The *picalm1a;1b;4a;4b* mutant displays a dwarf phenotype, which is alleviated in the presence of CA-YDA (+/-), indicating that activation of the YDA-MAPK pathway can compensate for the growth defects caused by loss of PICALM function. Scale bar: 3 cm.

(**B**) Quantification of plant height for the indicated genotypes. Statistical analysis was performed using one-way ANOVA followed by Tukey's HSD test, with significant differences (p-values) noted for specific genotype comparisons (n = 12 independent plants).

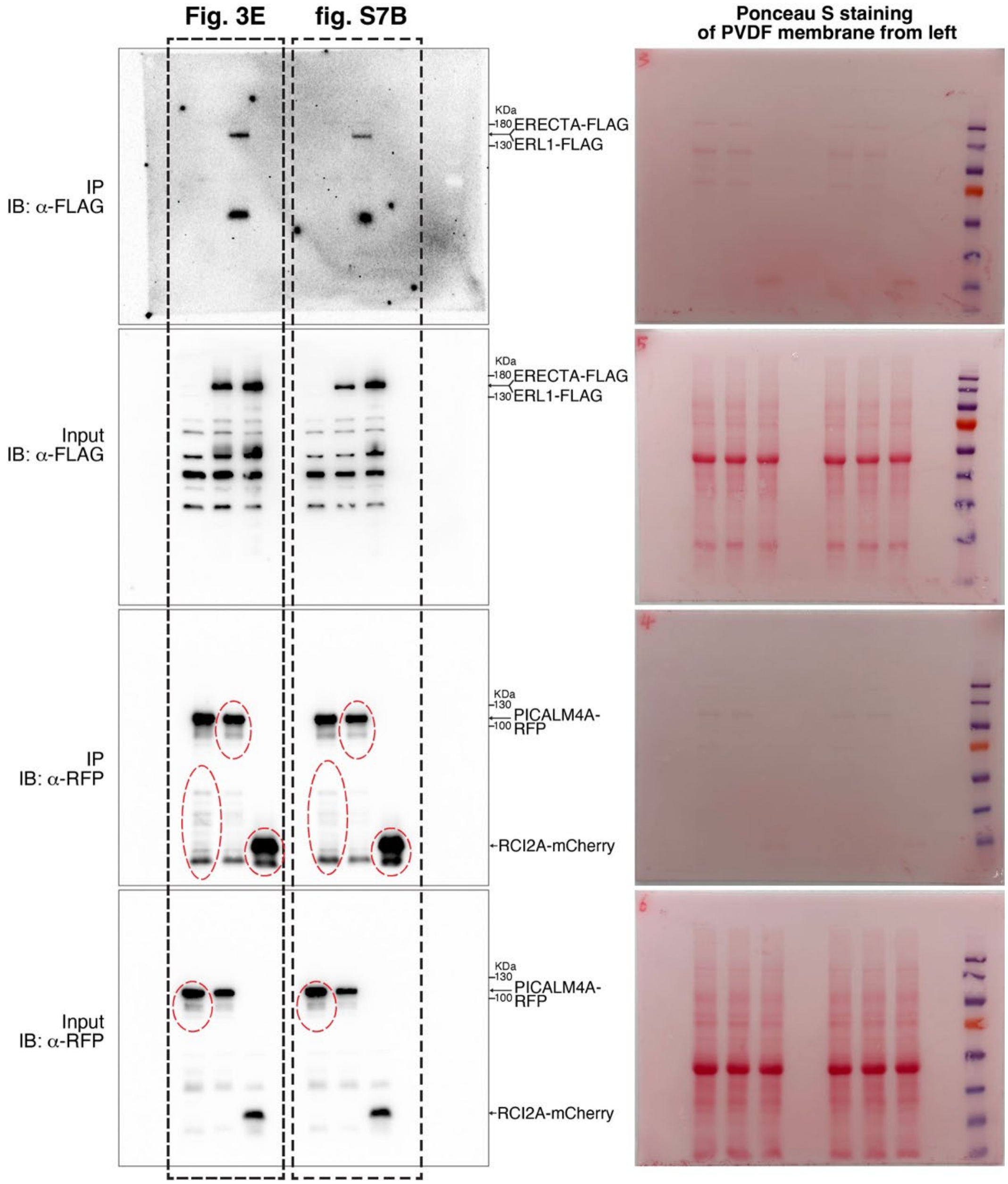

Fig. S13. Original uncropped immunoblot images corresponding to Fig. 3E and fig. S7B. Left panels show uncropped immunoblot images used for the Co-IP assay presented in Fig. 3E and fig. S7B. Rectangular dashed boxes indicate the cropped regions shown in the corresponding main and supplementary figures. Red dashed circles highlight the distinct signatures from two different figures. Right panels show Ponceau S staining of the total proteins on corresponding membranes.

Table S1. List of plasmids used in this study

Plasmid ID	Insertion	Vector	Bacterial selection	Plant selection
pPFB05	gERECTA-3xHA-TurboID with TAA	pENTR D-TOPO	Kanamycin	N/A
pPFB11	ERECTApro-gERECTA-3xHA-TurboID	R4pGWB501	Spectinomycin	Hygromycin
pPFB29	gLti6B-3xHA-TurboID	pENTR D-TOPO	Kanamycin	N/A
pPFB34	ERECTApro-gLti6B-3xHA-TurboID	R4pGWB501	Spectinomycin	Hygromycin
Stomata77	PICALM3pro:PICALM3-TagRFP	pGWB559	Spectinomycin	Hygromycin
Stomata78	PICALM4apro:PICALM4a-mRFP	pGWB1	Spectinomycin	Hygromycin
pPFB287	gPICALM1A-EGFP	pYBA-1132	Kanamycin	N/A
pPFB288	gPICALM1B-EGFP	pYBA-1132	Kanamycin	N/A
pPFB289	gPICALM2A-EGFP	pYBA-1132	Kanamycin	N/A
pPFB290	gPICALM2B-EGFP	pYBA-1132	Kanamycin	N/A
pPFB291	gPICALM3-EGFP	pYBA-1132	Kanamycin	N/A
pPFB292	gPICALM4A-EGFP	pYBA-1132	Kanamycin	N/A
pPFB293	gPICALM4B-EGFP	pYBA-1132	Kanamycin	N/A
pPFB294	gPICALM5A-EGFP	pYBA-1132	Kanamycin	N/A
pPFB295	gPICALM5B-EGFP	pYBA-1132	Kanamycin	N/A
pPFB296	gPICALM6-EGFP	pYBA-1132	Kanamycin	N/A
pPFB285	UBQ10 promoter (-1986bp to -1)	pENTR5'-TOPO	Kanamycin	N/A
pPFB298	gPICALM3	pKUT612 D-TOPO	Kanamycin	N/A
pPFB319	UBQ10pro_PICALM3-EYFP	R4pGWB540	Spectinomycin	Hygromycin
pPFB299	gPICALM4A	pKUT612 D-TOPO	Kanamycin	N/A
pPFB320	UBQ10pro_PICALM4A-EYFP	R4pGWB540	Spectinomycin	Hygromycin
Stomata87	ERECTA cytoplasmic region 604-976aa	pENTR D-TOPO	Kanamycin	N/A
Stomata88	PICALM1A CDS	pENTR D-TOPO	Kanamycin	N/A
Stomata89	PICALM2B CDS	pENTR D-TOPO	Kanamycin	N/A
Stomata90	PICALM3 CDS	pENTR D-TOPO	Kanamycin	N/A
Stomata91	PICALM4A CDS	pENTR D-TOPO	Kanamycin	N/A
Stomata92	PICALM4B CDS	pENTR D-TOPO	Kanamycin	N/A
Stomata93	PICALM5A CDS	pENTR D-TOPO	Kanamycin	N/A
Stomata94	PICALM6 CDS	pENTR D-TOPO	Kanamycin	N/A
Stomata95	PICALM8 CDS	pENTR D-TOPO	Kanamycin	N/A
Stomata96	PICALM9B CDS	pENTR D-TOPO	Kanamycin	N/A
Stomata97	PICALM10B CDS	pENTR D-TOPO	Kanamycin	N/A
Stomata98	ERECTA cytoplasmic region 604-976aa	pEU-E01-AGIA-GW-STOP	Ampicillin	N/A
Stomata99	PICALM1A CDS	pEU-E01-GW-bls-STOP	Ampicillin	N/A
Stomata100	PICALM2B CDS	pEU-E01-GW-bls-STOP	Ampicillin	N/A
Stomata101	PICALM3 CDS	pEU-E01-GW-bls-STOP	Ampicillin	N/A
Stomata102	PICALM4A CDS	pEU-E01-GW-bls-STOP	Ampicillin	N/A
Stomata103	PICALM4B CDS	pEU-E01-GW-bls-STOP	Ampicillin	N/A
Stomata104	PICALM5A CDS	pEU-E01-GW-bls-STOP	Ampicillin	N/A
Stomata105	PICALM6 CDS	pEU-E01-GW-bls-STOP	Ampicillin	N/A
Stomata106	PICALM8 CDS	pEU-E01-GW-bls-STOP	Ampicillin	N/A
Stomata107	PICALM9B CDS	pEU-E01-GW-bls-STOP	Ampicillin	N/A
Stomata108	PICALM10B CDS	pEU-E01-GW-bls-STOP	Ampicillin	N/A

Table S2. List of oligo DNA primers used in this study

Primer ID	Sequence	Description
Bai335_PICALM3_gDNA_F_KUT612_Xho1	CGCGGCCGCACTCGAGATGTCAGGTGGTGGTGGATCACAG	Cloning
Bai336_PICALM3_gDNA_R_KUT612_BamH1	GCCCTCTAGAGGATCCCATATACCTTGGATATGGGTTGTAACCTTGC	Cloning
Bai337_PICALM4A_gDNA_F_KUT612_Xho1	CGCGGCCGCACTCGAGATGGCTCCGAGTATTCGAAAAGCG	Cloning
Bai338_PICALM4A_gDNA_R_KUT612_BamH1	GCCCTCTAGAGGATCCGTAAGGATTGTTGTAGTAATACCCCGTCG	Cloning
Bai339_PICALM4B_gDNA_F_KUT612_Xho1	CGCGGCCGCACTCGAGATGGCGCTAAGCATGCGAAAAGCG	Cloning
Bai340_PICALM4B_gDNA_R_KUT612_BamH1	GCCCTCTAGAGGATCCGTAAGGGTTGTTGTAGTAATAACCCATCGG	Cloning
PICALM3_1132-F	GGAGAGGACAATTGGAGCTCATGTCAGGTGGTGGTGGATCACAG	Cloning
PICALM3_1132-R	CCCTTGCTCACCATGGTACCCATATACCTTGGATATGGGTTGTAAC	Cloning
PICALM4A_1132-F	GGAGAGGACAATTGGAGCTCATGGCTCCGAGTATTCGAAAAGCG	Cloning
PICALM4A_1132-R	CCCTTGCTCACCATGGTACCGTAAGGATTGTTGTAGTAATACCCCG	Cloning
PICALM4B_1132-F	GGAGAGGACAATTGGAGCTCATGGCGCTAAGCATGCGAAAAGCG	Cloning
PICALM4B_1132-R	CCCTTGCTCACCATGGTACCGTAAGGGTTGTTGTAGTAATAACC	Cloning
Bai-020-HA-TurboID-F2	AGGCGCGCCTACCCGTATGATGTTCCGGATTACGCTGGCTA	Cloning
Bai-023-TurboID-R1	AGGCGCGCCcTTACTTTTCGGCAGACCGCAGAC	Cloning
Bai120-Lti6B-F	GCCGCCCCCTTCACCATGAGTACAGCCACTTTCGT	Cloning
Bai121-Lti6B-R	GGCGCGCCCACCCTTCTTGGTGATGATATAAAGAGCGT	Cloning
ERECTA_endo f	GCCCCCTTCACCATGCCGCATAATCCTCCTCCT	Cloning
ERECTA_r	GCGCCCACCCTTACTCACTGTTCTGAGAAATAACT	Cloning
PICALM1a TOPO fw	caccATGGGAACGCTACAGTCATGG	Cloning
PICALM1a no stop rv	AATGAGGCCAGTGCTACGGAAC	Cloning
PICALM4b-f	CACCATGGCGCTAAGCATGCG	
PICALM4b-sal1-r	GCGTCGACTCAGTAAGGGTTGTTGTAGTAAT	
PICALM8-f	GCCCCCTTCACCATGAGGCTTGATTTATCTGCC	Cloning
PICALM8-stop-r	GCGCCCACCCTTACGTTCATATGCTTTCTCTGT	Cloning
PICALM9b_CDS_for	caccATGAAGCTGTGGAAACGAGCTG	Cloning
PICALM9b_CDS-sc_rev	GAATGTAATCAAATCTGGCATTGTATAAGTG	Cloning

PICALM10b-f	GCCCCCTTCACCATGCCAGGACTCAAAACTC	Cloning
PICALM10b-stop-r	GCGCCCACCCTTGAAATCAGAATCGTCGAATGTT	Cloning
PICALM2b-stopr	GCGCCCACCCTTGAGGAGCATATGATGATGGTT	Cloning
PICALM3-stopr	GCGCCCACCCTTCATATACCTTGGATATGGGTTGT	Cloning
PICALM4a-stop r	GCGCCCACCCTTGTAAGGATTGTTGTAGTAATACC	Cloning
PICALM4b-stop	GCGCCCACCCTTGTAAGGGTTGTTGTAGTAATAAC	Cloning
PICALM5a-stop r	GCGCCCACCCTTATATTGAGGTGTGTAAGAGTAAG	Cloning
PICALM6-stop r	GCGCCCACCCTTACTCAAGTGCTTGGCTATGA	Cloning
picalm1a_LP	TTTGTTCAATTTTCCCCACAG	Genotyping
picalm1a_RP	ACCATAACCGGAGGTATTTGG	Genotyping
picalm1b_LP	GGTGTATCATCATGCAGACCC	Genotyping
picalm1b_RP	TCTTTGATGTCATTTCCCGAG	Genotyping
picalm2a_LP	GCCAGGTCACTGGTAGTTGT	Genotyping
picalm2a_RP	ACAATCCCACGCTGCATCAA	Genotyping
picalm2b_LP	CTTTTACCTGTTGGCCAGCTC	Genotyping
picalm2b_RP	TAGGATTTGGATGTTGCGATC	Genotyping
picalm3_LP	AAATCAGTTGTCGAATGTGGC	Genotyping
picalm3_RP	TTGATATGCAACGGAATGATG	Genotyping
picalm4a_LP	ATCTCACAACGCCAAATTTTG	Genotyping
picalm4a_RP	TCTCATCTCTCCCTCCTCCTC	Genotyping
picalm4b_LP	TTGGCAAAAATAATTATGCCTG	Genotyping
picalm4b_RP	GAGAAGATGATGTCTCTGGCG	Genotyping
picalm5a_LP	GGTAAGGCCTTTATTGCGTTC	Genotyping
picalm5a_RP	CTCGTCACGAAGAGTTTCCAG	Genotyping
picalm5b_LP	CATTCATTTGCACGTATGCTG	Genotyping
picalm5b_RP	TAATTTCCTTGGAACACACGC	Genotyping
ERECTA_RH436f_qRT	TGGAAAATGGTAGCCTCTGG	RT-qPCR
ERECTA_RH437r_qRT	CTTAAGCCGTGTGTCCCAAT	RT-qPCR
YFP_f2_qRT	ATCATGGCCGACAAGCAGAA	RT-qPCR
YFP_r2_qRT	TCTCGTTGGGGTCTTTGCTC	RT-qPCR
PICALM4A_f3_qRT	CAAGTTTGCGCTTGCACTCT	RT-qPCR
PICALM4A_r3_qRT	CAAGCAGAGGTCACTCCGTT	RT-qPCR
ACTIN2-F_qRT	GATGAGGCAGGTCCAGGAATC	RT-qPCR
ACTIN2-R_qRT	AACCCCAGCTTTTTAAGCCTTT	RT-qPCR

Movie S1. (Separate file) Live-cell imaging reveals dynamic trafficking of ERECTA-YFP with PICALM4A-RFP in BFA bodies of Arabidopsis epidermal cells. Two frames per second. Scale bar: 10 μm.

Dataset S1. (Separate file) Comparative ERECTA proteomics by TbID-PL and ET-AP using *er-105* and ERECTApro::Lti6B-HA-TbID controls.

Dataset S2. (Separate file) Comparative proteomics of ERECTA-TbID-PL with and without biotin treatment

Dataset S3. (Separate file) Comparative proteomics of ERECTA-TbID-PL from microsomal and cytosolic compartments

Dataset S4. (Separate file) Subcellular localization prediction of ERECTA-TbID-PL candidates based on SUBA5 annotations

Dataset S5. (Separate file) Statistical source data for all figures
